# Five years’ trajectories of functionality and pain in patients after hip or knee replacement and association with long-term patient survival

**DOI:** 10.1038/s41598-020-71277-3

**Published:** 2020-09-01

**Authors:** Stefan Repky, Gisela Büchele, Klaus-Peter Günther, Klaus Huch, Hermann Brenner, Til Stürmer, Jan Beyersmann, Rolf E. Brenner, Dietrich Rothenbacher

**Affiliations:** 1 Institute of Statistics, Ulm University, Ulm, Germany; 2 Institute of Human Genetics, Ulm University, Ulm, Germany; 3 Institute of Epidemiology and Medical Biometry, Ulm University, 89081 Ulm, Germany; 4 University Center of Orthopaedics and Traumatology, University Medicine Carl Gustav Carus Dresden, TU Dresden, Dresden, Germany; 5 Birkle Clinic, Department of Orthopedics and Trauma Surgery, Bodenseeklinik, Überlingen, Germany; 6 Division of Clinical Epidemiology and Aging Research, German Cancer Research Center (DKFZ), Heidelberg, Germany; 7 Network Aging Research, University of Heidelberg, Heidelberg, Germany; 8 Department of Epidemiology, Gillings School of Global Public Health, University of North Carolina at Chapel Hill, Chapel Hill, NC USA; 9 Department of Orthopedics, Division for Biochemistry of Joint and Connective Tissue Diseases, Ulm University, Ulm, Germany; 10 Centre for Trauma Research, Ulm University, Ulm, Germany

**Keywords:** Rheumatology, Epidemiology

## Abstract

To describe the 5 years’ trajectories in functionality and pain of patients with hip or knee osteoarthritis and arthroplasty and analyze the association of these with long-term patients survival. Patients with OA receiving total hip or knee arthroplasty were recruited and completed two sets of standardized questionnaires for functionality and pain 6, 12, and 60 months postoperatively. Multivariate mixed models were conducted to assess trajectories over time and the resulting improvement per month during the last time period was included in a landmark-model to estimate adjusted hazard ratios for mortality. In total 809 patients with joint replacement were included (mean age 65.0 years, 62.2% female), 407 patients died (median follow-up 18.4 years). Both instruments of functionality and pain showed extensive improvement during the first 6 months. Baseline and change in functionality (both p < 0.001) and pain (p = 0.02) during the first 6 months were associated with mortality. Better values in functionality corresponded with improved survival whereas the association with the pain scores was inverse. In patients with hip and knee OA, an explicit improvement in function is seen within the first 6 months after arthroplasty. In addition, especially the functionality scores at baseline as well as their improvement showed an association with long-term patient survival.

## Introduction

Osteoarthritis (OA) is a very common musculoskeletal disorder. Prevalence of OA is increasing with age and OA leads to functional disability and pain in the joints^1,2^. Arthroplasty is an established treatment in symptomatic patients in order to restore functionality, relief pain, and increase overall quality of life. It prevents disability and also enables a renewed physically active lifestyle, which is an important component of health^3^.

Overall, patients with OA seem to have no increased all-cause mortality compared to the general population, also previous studies revealed inconsistent results^4^. A recent meta-analysis which investigated the association of symptomatic or radiological OA and all-cause mortality came to the conclusion that so far no reliable evidence exists for an association of OA with general and all-cause mortality^5^. We came to a similar conclusion when analyzing the 20 years’ mortality after the first hip or knee joint replacement in our study population^6^. However, as impaired function and chronic pain associated with OA may result in disability as well as in decreased physical activity, these patients may also have long-term consequences on a variety of health-related endpoints, and finally on long-term survival. However, studies that investigated the trajectories of functionality and pain after arthroplasty and the association with long-term health consequences are scarce yet. Additionally, the difference in association with long-term mortality between generic and osteoarthritis-specific instruments has not been investigated so far.

Therefore, the aim of this investigation was to describe the 5 years’ trajectories in functionality and pain of patients from the Ulm Osteoarthritis Study cohort, who underwent a hip or knee replacement due to osteoarthritis and were characterized with different measurement instruments of pain and functionality. The resulting trajectories have then been analyzed to investigate the association with long-term patient survival after adjustment for multiple covariates.

## Methods

### Study design and study population

For this prospective cohort study patients for unilateral total hip or knee arthroplasty due to advanced OA were recruited consecutively between January 1995 and December 1996 in four hospitals in the South-West of Germany. The inclusion criteria (i.e. white, age not exceeding 75 years, absence of malignancies, inflammatory diseases, or corticosteroid medication; no previous joint replacement) were fulfilled by N = 809 patients who also gave written informed consent. The initial study (details in^7,8^), as well as the current follow-up, was approved by the Ethics Committee of Ulm University (No. 164/14). The study was conducted according to the relevant guidelines and regulations and the Declaration of Helsinki.

### Study data collection and classification

Among others, detailed information about comorbidities, symptoms, and medical history as well as demographic data, was gathered by standardized interviews. We characterized “secondary OA” by the following reasons: infection, avascular necrosis, and osteochondritis, hemorrhagic diathesis, traumatic events with radiologically and/or surgically confirmed structural joint lesions as well as sequelae of slipped femoral capital epiphysis and acetabular dysplasia in pelvic radiographs. An idiopathic origin of “primary OA” was assumed in the absence of these risk factors. Missing values in secondary OA were handled as separate category “unknown”. Serum cholesterol levels and serum uric acid levels were obtained^9,10^ in non-fasting serum-samples taken preoperatively by standard venipuncture.

### Assessment of functionality and pain

Different assessments were applied for functionality and pain. To measure functionality, the Hannover Functionality Status Questionnaire (FFbH) and the functionality sub-scale of the Western Ontario and McMaster University Osteoarthritis Index (WOMAC) were applied. The basic version of FFbH (including 18 single questions regarding the last seven days) was used to assess general function. WOMAC (including 17 single questions) was used to specifically describe function in the operated joint at the time point of assessment.

**Figure 1:**
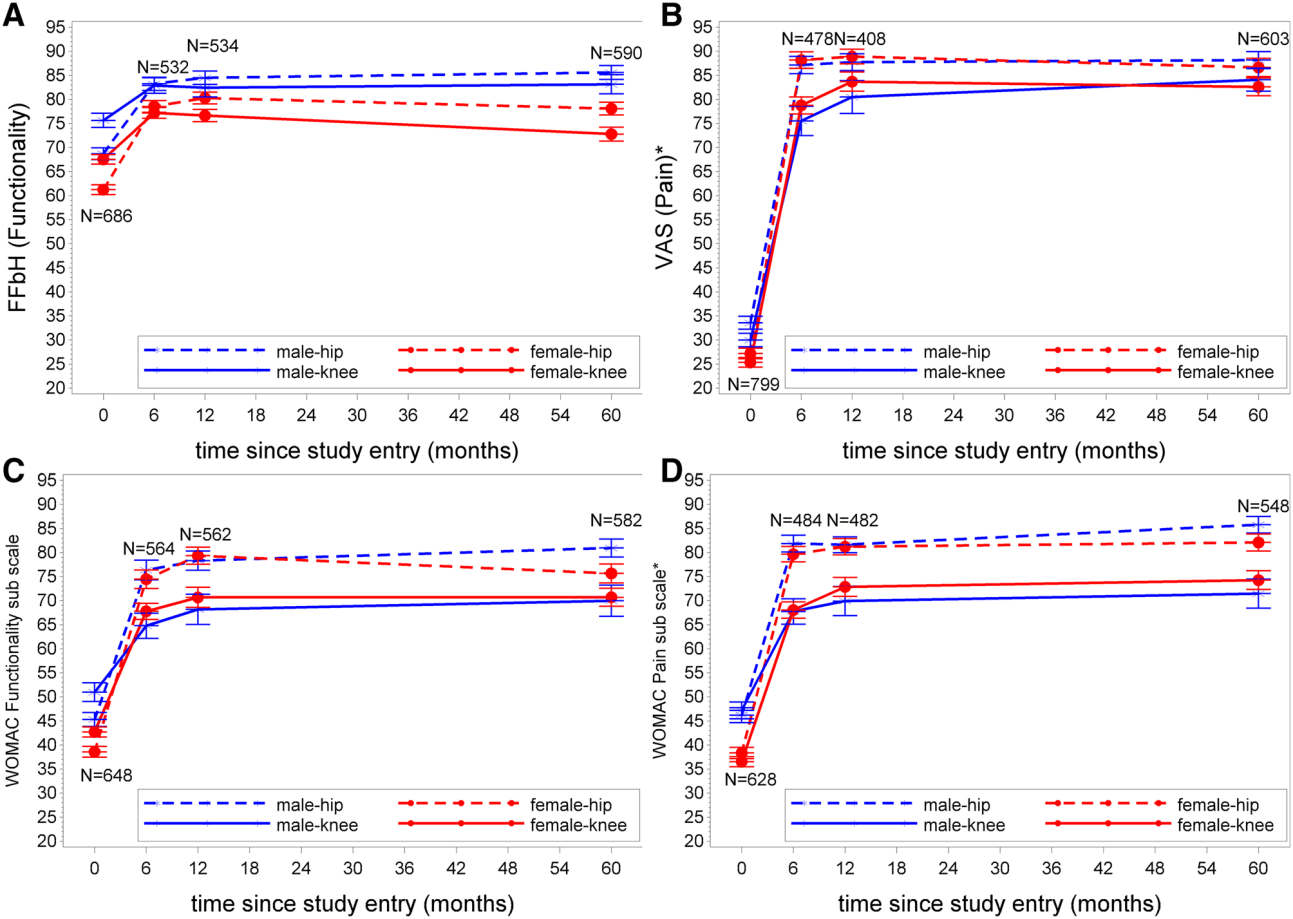
Trajectories of functionality (**A**,**C**) and pain (**B**,**D**) until the 60 months’ Follow-up. Shown values are means with standard errors. *FFbH* Hannover Functionality Status Questionnaire, *VAS* visual analogue scale, *WOMAC* Western Ontario and McMaster University Osteoarthritis Index, *N* number of pat. *Due to normalization, high scores equal less pain.

To measure pain, the Visual Analogue Scale (VAS) and the pain sub-scale of the WOMAC were applied. Both measures describe pain in the operated joint at the moment of assessment, the WOMAC pain sub-scale, however, concentrates on typical OA-associated circumstances. For comparability, each score was transformed onto a scale from 0 to 100 with higher values being positive for a patient (better functionality, less pain). The measurements of these instruments were applied at baseline as well as at 6 months, 12 months, and at 60 months. Missing values in assessments occurred due to loss to follow-up of patients or missed follow-up visits during the study course. The exact number of observations is displayed in Fig. 1A–D.

### Assessment of mortality

Mortality was assessed during the follow-up time up to 20 years via residents' registration office. In case of death, the exact date of death was obtained from the respective residents' registration office. Information was gathered up until 31st August 2015. Further details can be found elsewhere^6^.

### Statistical analysis

The main characteristics of the study population at baseline were described. The selection of characteristics included in these analyses was based on a previous investigation of the same dataset^6^. The selected characteristics were age, gender, diabetes, cholesterol, uric acid, heart insufficiency, hypertension, overweight, smoking status, localization of OA, and secondary OA as covariates for overall survival (details of variable selection see^6^).

Two different approaches were used to analyze both, a functionality value and a pain value in one statistical model. Approach 1 combined the FFbH functionality score with the VAS pain score. Approach 2 combined the two sub-scales for functionality and pain of the WOMAC. To assess the course of functionality and pain, the mean values of all assessments with corresponding standard errors were computed for baseline and follow-up time points. The resulting mean courses of all four assessments were plotted. Correlations between measured baseline values and trajectories (changes within specific time periods) were assessed by Pearson’s correlation coefficients for descriptive purposes.

The statistical analysis was conducted via a landmark Cox model combined with a prior, two-dimensional linear mixed effects model. We excluded observations with missing baseline measurements in pain or functionality for this analysis. The linear mixed effects model was used to estimate the partwise-linear trajectories of pain and functionality in the corresponding landmark dataset. The linear mixed effects model was calculated including no covariates other than time as predictor, using the breakpoints 6 and 12 months after follow-up. Then, the estimated current slopes as well as baseline functionality, pain and other baseline characteristics were included in the landmark Cox model^11–13^. Due to the follow-up times, landmark times were chosen to be 6, 12 and 60 months after follow-up and the previously described Cox model was calculated for each landmark time. For further information concerning the exact statistical methods see the Supplementary Material. Hazard ratios (HR) were estimated for the improvement in functionality or pain. The HRs with 95% confidence intervals (CI) and additional p values are shown for all 6 resulting sub-models (3 time points for 2 approaches). No adjustment for multiple testing was performed. All analyses were performed using SAS 9.4 (SAS Institute Inc., Cary, NC, USA).

## Results

In the baseline investigation, 809 patients with OA were included; of these, 389 patients had arthroplasty of the knee, and 420 patients had arthroplasty of the hip, respectively. As displayed in Table 1 median age of patients with knee OA was 5 years older compared to patients with hip OA. Patients with knee OA also exhibited more comorbidities. In the subsets of patients with available assessments of functionality and pain, the described baseline characteristics were comparable to the population without missing values (data not shown). At the end of the 20 years’ follow-up (median observation time 18.4 years), N = 407 (50.3%) patients were deceased and n = 13 (1.6%) patients had been lost to follow-up.

**Table 1:** Characteristics of patients with osteoarthritis (OA) at baseline and 20 years’ follow-up.

At baseline	Total (N = 809)	Male (N = 305)		Female (N = 504)	
		Localization		Localization	
		Hip (N = 199)	Knee (N = 106)	Hip (N = 221)	Knee (N = 283)
Age (years), median (IQR)	65.0 (58.0; 70.0)	59.0 (54.0; 66.0)	65.0 (60.0; 69.0)	64.0 (56.0;70.0)	68.0 (64.0;71.0)
Smoker, N (%)	102 (12.6%)	45 (22.6%)	20 (18.9%)	25 (11.3%)	12 (4.2%)
Former smoker, N (%)	238 (29.4%)	96 (48.2%)	50 (47.2%)	45 (20.4%)	47 (16.6%)
Secondary OA, N (%)	296 (36.6%)	81 (40.7%)	49 (46.2%)	89 (40.3%)	77 (27.2%)
History of overweight/obesity, N (%)	466 (57.6%)	85 (42.7%)	64 (60.4%)	106 (48.00%)	211 (74.6%)
Diabetes mellitus type 2, N (%)	70 (8.7%)	15 (7.5%)	11 (10.4%)	13 (5.9%)	31 (11.00%)
Heart insufficiency, N (%)	153 (18.9%)	21 (10.6%)	14 (13.2%)	36 (16.3%)	82 (29.1%)
Hypertension, N (%)	415 (51.3%)	94 (47.2%)	54 (50.9%)	96 (43.4%)	171 (60.4%)
Cholesterol (mmol/l), median(Q1;Q3), (N = 683)	5.7 (5.1 ;6.4)	5.6 (5.0; 6.1)	5.6 (5.0; 6.2)	5.7 (5.1; 6.6)	5.9 (5.2; 6.5)
Uric Acid (mmol/l), median (Q1;Q3), (N = 699)	315.4 (265.0; 376.0)	351.1 (303.4; 411.0)	351.1 (296.0; 404.6)	289.5 (240.0; 330.0)	297.5 (243.9; 368.9)
**20 years’ follow-up**					
Deaths, N (%)	407 (50.3%)	89 (44.7%)	68 (64.2%)	81 (36.7%)	169 (59.7%)
Observation time, median (Q1;Q3)	18.36 (11.38; 19.20)	18.54 (10.09; 19.28)	14.44 (9.61; 18.99)	18.67 (13.45; 19.31)	16.93 (11.45; 19.17)
Mortality rate per 1,000 patient years, rate(CI)	33.8 (30.5; 37.1)	30.2 (23.9; 36.4)	47.3 (36.0; 58.5)	23.5 (18.3; 28.6)	40.5 (34.4; 46.6)

*IQR(Q1;Q3)* interquartile range, *N* number of patients, *CI* confidence interval.

### Trajectories of functionality and pain

Score values over time of functionality (FFbH and WOMAC sub-scale functionality) and of pain (VAS and WOMAC sub-scale pain) are displayed in Fig. 1A–D stratified for localization of OA and gender. Patients started at relatively low levels of functionality and with high levels of pain at baseline. They experienced substantial improvements during the first 6 months after arthroplasty, in FFbH as well as in the VAS, and also in both WOMAC sub-scales. While looking at the differences between patients with knee OA and patients with hip OA one must keep in mind that knee-patients were older than hip patients. Further details are provided in Supplementary Table S1. Baseline values and trajectories of the FFbH and VAS-pain scale were only moderately correlated. In contrast, the WOMAC sub-scales were strongly correlated (Supplementary Table S2).

Functionality measured by FFbH was in general lower (i.e. worse functionality) in knee OA compared to hip OA and in females compared to males at baseline (Fig. 1, panel A). It increased substantially until month 6 and thereafter decreased until month 60. In general, patients with hip OA had higher functionality values (i.e. better functionality) than patients with knee OA, this was also the case for WOMAC functionality. Within gender, patients with hip OA had better values than knee OA during follow-up. VAS pain values showed an even stronger increase from point zero to 6 months’ follow-up, and patients with hip OA showed higher (less pain) values (Fig. 1, panel B). Patients with hip OA reached a higher plateau than patients with knee OA and showed no decrease after 12 months.

The WOMAC functionality sub-scale showed a strong increase up to month 6 and less steep to month 12, and was also reaching higher levels in patients with hip OA compared to knee OA (Fig. 1, panel C). Both sites and genders showed no further notable changes after month 12 until month 60. A similar pattern was found for the WOMAC pain sub-scale (Fig. 1, panel D).

### Association of functionality and pain with survival-approach 1 (FFbH and VAS)

Higher values of FFbH and therefore better functionality at baseline were associated with lower overall mortality [HR at landmark 6 months 0.98 (95% CI 0.97–0.99) per point increase]. This association remained statistically significant also in landmark times 12 and 60 months (Table 2, upper panel). Even 60 months later better baseline FFbH values are still associated with lower subsequent mortality of patients [HR = 0.98 (95% CI 0.97–0.99)]. Additionally, the improvement in FFbH over the first six months was also associated with lower mortality (HR = 0.80, 95% CI 0.71–0.90). The HRs of the 12 months’ and 60 months’ landmark times tended into the same direction.

**Table 2:** Association of functionality and pain with long-term mortality after 20 years of follow-up by landmark Cox proportional hazard models.

Variables	Landmark time 6 months		Landmark time 12 months		Landmark time 60 months	
	Hazard ratio (CI)	p value	Hazard ratio (CI)	p value	Hazard ratio (CI)	p value
**Approach 1: FFbH functionality and VAS pain**						
FFbH at baseline	**0.98 (0.97; 0.99)**	** < .0001**	**0.99 (0.98; 0.99)**	**0.0017**	**0.98 (0.97; 0.99)**	**0.0021**
Improvement per month in FFbH	**0.80 (0.71; 0.90)**	**0.0002**	0.76 (0.56; 1.01)	0.0627	0.50 (0.13; 1.86)	0.30
VAS at baseline	1.01 (0.99; 1.02)	0.12	1.00 (0.99; 1.01)	0.74	1.00 (0.99; 1.01)	0.68
Improvement per month in VAS	**1.06 (1.01; 1.12)**	**0.0230**	1.04 (0.96; 1.14)	0.32	1.06 (0.74; 1.51)	0.75
**Approach 2: WOMAC sub-scales for functionality and pain**						
WOMAC functionality Score at baseline	1.00 (0.99; 1.01)	0.85	1.00 (0.99 ; 1.01)	0.96	1.00 (0.99 ; 1.01)	0.93
Improvement per month in WOMAC functionality score	0.93 (0.83 ; 1.05)	0.24	0.99 (0.9 ; 1.09)	0.82	1.41 (0.44 ; 4.52)	0.57
WOMAC pain score at baseline	1.00 (0.99 ; 1.00)	0.78	1.00 (0.99 ; 1.00)	0.33	1.00 (0.99 ; 1.00)	0.32
Improvement per month in WOMAC pain score	1.01 (0.99 ; 1.04)	0.39	1.00 (0.98 ; 1.02)	0.85	0.91 (0.70 ; 1.18)	0.47

Results with p values less than 0.05 were marked as significant (bold).

This method allows to estimate the survival probability after the respective landmark time point (is then begin of observation time for survival) conditional to the information of pain and function from baseline and also including simultaneously the change since the last landmark point (e.g. at landmark 12 months the change since landmark 6 months) after additional adjustment for covariates. The results within one approach are adjusted for each other (functionality and pain simultaneously as well as for the baseline value and improvement values within one landmark time point) and the respective covariates.

All models adjusted for age, gender, diabetes, cholesterol, uric acid, heart insufficiency, hypertension, overweight, smoking status, localization of OA, and secondary OA.

*WOMAC* Western Ontario and McMaster University Osteoarthritis Index, *FFbH* Hannover Functionality Status Questionnaire, *CI* confidence interval, *VAS* visual analogue scale.

The VAS pain baseline values showed no association with mortality. However, more pronounced improvement in pain during the first 6 months was associated with increased mortality [HR = 1.06 (95% CI 1.01–1.12)].

### Association of functionality and pain with survival-approach 2 (WOMAC sub-scales)

The analyses using the osteoarthritis-specific WOMAC sub-scales to quantify functionality and pain yielded no associations with overall mortality (Table 2, lower panel). Both baseline variables resulted in HR estimates whose 95% CIs included the null-effect value in all cases.

## Discussion

In this cohort study of 809 patients with arthroplasty of hip or knee, we found a clear improvement in both functionality and pain after arthroplasty within the first 6 months which persisted, although less steep, until month 12. Better FFbH function scores at baseline, as well as improvement during the landmark times of 6, 12, and 60 months, were associated with improved survival during 20 years of follow-up. Notably, none of these associations with mortality were evident with the osteoarthritis specific WOMAC sub-scales pain and functionality primarily focusing on OA-specific aspects. Therefore trajectories of pain and function related to factors associated with general health aspects may be more important for long-term survival than joint-specific trajectories within a period of 1 year after arthroplasty.

Previous studies already showed an increase in functionality as well as a relief in pain after hip or knee arthroplasty with more pronounced effect in the hip^14–16^. A study by Lenguerrand et al. found that the main improvement in pain, as well as functionality, takes place in patients with hip and knee arthroplasty during the first three months after arthroplasty^17^, whereas no further changes were evident in the following nine months. Dowsey, Smith, and Choong tried to model the pain and functionality of patients with knee arthroplasty up to 5 years after operation^18^. Their measurements were taken once a year and showed an improvement during the first year in all subclasses (except patients with high pain scores) and approximately constant levels in later years. Our results are in line with the findings of these studies and expand them in several important respects.

Looking on specific factors Nüesch et al. reported that mortality in patients with hip or knee OA is linked to walking disability^19^. Hawker et al. confirmed this observation in patients with hip or knee OA who were followed over 13 years and reported an association of all-cause mortality with the WOMAC function sub-scale and walking disability but not with pain^20^. Interestingly, in the subgroup which received joint arthroplasty a reduced risk for cardiovascular events and improved patient survival was identified going in line with improved physical function. Joint arthroplasty also reduces chronic pain which may carry itself an increased risk for mortality. In a large cohort study from Denmark, patients with chronic pain had a 39% increased risk of death compared to the general population^21^. Management of peri- and postoperative pain is an important issue in patients with arthroplasty of the hip or knee. Several studies that investigated the effect of different strategies emphasized the importance of adequate pain management to prevent persistent postoperative pain^15,22,23^. Dumenci et al.^24^ estimated that among adults after knee arthroplasty about 18% had persistent pain, poor function, or both after 12 months. Interestingly, a recent review and meta-analysis suggested an overall modest risk of chronic pain in general with high heterogeneity between studies^25^. This variation was partly attributed to different pain phenotypes included. More recent studies on pain and mortality in adults above 50 years of age indicated that mainly pain which compromises daily life activities is associated with an increased risk of mortality^26^ and that functional limitation, as well as physical inactivity, may represent relevant mediators^27^.

Arthroplasty of hip or knee usually improved cardiovascular fitness in patients after hip arthroplasty investigated up to 2 years postoperatively^28^, an accepted predictor for all-cause mortality and cardiovascular events^29^. Interestingly, disability but not OA itself has recently been claimed to predict cardiovascular disease in a population-based cohort^30^.

However, currently there is no good evidence to support the association between joint replacement and increased physical activity. Patient self-reported physical activity seems neither accurate nor reproducible as assessed in a group of patients pre-operatively and 6–8 weeks post-operatively^31^. A study conducted with sensor-based measurements in 63 patients awaiting hip or knee arthroplasty from Australia showed no change to measurements 6 months later^32^, also 6 months measurements may still be influenced by post-operative pain and complications. In another study from Australia including 51 patients with total hip arthroplasty also no improvement in sensor-measured 24-h activity profiles at six months was seen^33^. A review including eight studies with patients with hip or knee arthroplasty and also sensor-based measurements showed negligible changes at 6 months follow-up, but larger changes at 12 months, which were especially evident for hip OA^34^. Still existing studies are based on relatively small numbers and partly suffer a high risk of bias. Therefore larger and better designed studies are still needed to make firm conclusions.

Our results indicate that long-term mortality of OA patients after joint replacement is more associated with the general health status, physical activity, and comorbidities than with joint-specific impairment^35^. Therefore, joint replacement should not be performed too late. Further investigations should look especially into the primary determinants and long-term consequences of different functional and pain trajectories especially visible in the first 6 months after OA. It seems plausible that the time shortly after OA is an important time slot for long-term prognosis, still with the possibility to eventually modifying treatment and rehabilitation efforts.

The study has the following strength: the study population with more than 800 patients with OA and arthroplasty of hip and knee was very well characterized at baseline and had a well-defined disease status. In addition, different measurements for functionality and pain were available during follow-up: on the one side a more general assessment of functionality by the FFbH score and of pain by the VAS, on the other side, an OA-specific assessment with the two WOMAC sub-scales.

The advanced landmark analyses used in this investigation extend standard landmark approaches due to the inclusion of a previous mixed model. By prior estimation of the trajectories, we were able to account for missing values occurring over the course of time as well as for a time-dependent bias (immortal time bias). A limitation of this technique is the disregard towards the joint distribution between survival process and trajectory^36–39^.

Another advantage is based on the design of landmarking itself. We were able to gain estimators, evaluating the association of the trajectory slopes at certain landmark times with survival. This is important because patients undergo greater changes during the first period after an arthroplasty. This period is therefore much more likely associated with survival than later ones. Another strength is that we were able to mutually adjust for functionality and pain. A limitation in our advanced approach arises due to the created linear dependencies between different estimations in our trajectory. Therefore, we cannot conclude with certainty, that the borderline significant results in a change of functionality included in the 12 months’ model for FFbH are due to an existing association or the dependency to the change at landmark time 6. A landmarking sensitivity analysis without mixed modeling did not find such an effect (results not shown). As an additional limitation, we did not have data from all patients for all the follow-up time points. In addition, we had a limited sample size especially in the subgroups and we were not able to assess the specific cause of death. Furthermore, it has to be considered that especially fitter patients may have benefitted more from arthroplasty and eventually survived longer.

Our final results suggest that in patients with hip and knee OA, arthroplasty leads to a clear improvement in both functionality measurements within the first 6 months, which seems to persist, although less steep, until month 12. The more pronounced improvement of the more generic VAS-pain score at the landmark time of 6 months showed an inverse association with long-term survival, pointing to a higher importance of general health status compared to joint-specific impairment for long-term patient survival.

## Supplementary information

Supplementary file1

Supplementary file2

## Data Availability

Due to ethical restrictions, the data cannot be made publicly accessible but are available upon reasonable request. The request should be directed to Prof. Rothenbacher (dietrich.rothenbacher@uni-ulm.de).
