# Supplementary material for: Five years’ trajectories of functionality and pain in patients after hip or knee replacement and association with long-term patient survival
Source: Sci Rep. 2020 Sep 1;10:14388. doi: 10.1038/s41598-020-71277-3 (PMC7463234; doi:10.1038/s41598-020-71277-3)
Supplement: Supplementary file 1 — Supplementary file1 [file 41598_2020_71277_MOESM1_ESM.docx]

**Five years’ trajectories of functionality and pain in patients after hip or knee replacement and association with long-term patient survival**

Stefan Repky, MSc^1,2#^, Gisela Büchele, PhD, MPH^3*^, Klaus-Peter Günther, MD^4^, Klaus Huch, MD^5^, Hermann Brenner, MD, MPH^6,7^, Til Stürmer, MD, MPH^8^, Jan Beyersmann PhD^1^, Rolf E. Brenner, MD^9^, Dietrich Rothenbacher, MD, MPH^3,10,^*

#equal contribution

^1^ Institute of Statistics, Ulm University, Ulm, Germany

^2^ Institute of Human Genetics, Ulm University, Ulm, Germany

^3^ Institute of Epidemiology and Medical Biometry, Ulm University, Ulm, Germany

^4^ University Center of Orthopaedics and Traumatology, University Medicine Carl Gustav Carus Dresden, TU Dresden, Dresden, Germany

^5^ Birkle Clinic, Department of Orthopedics and Trauma Surgery, Bodenseeklinik, Überlingen, Germany

^6^ Division of Clinical Epidemiology & Aging Research, German Cancer Research Center (DKFZ), Heidelberg, Germany

^7^ Network Aging Research, University of Heidelberg, Germany

^8^ Department of Epidemiology, Gillings School of Global Public Health, University of North Carolina at Chapel Hill, Chapel Hill, NC, USA.

^9^ Department of Orthopedics, Division for Biochemistry of Joint and Connective Tissue Diseases, Ulm University, Ulm, Germany.

^10^Centre for Trauma Research, Ulm University, Ulm, Germany

*Correspondence to:

Prof. Dr. med. Dietrich Rothenbacher, MPH
Institute of Epidemiology and Medical Biometry,

Ulm University

89081 Ulm - Germany

Telephone: 0049 731 50 31060

E-mail: [dietrich.rothenbacher@uni-ulm.de](mailto:dietrich.rothenbacher@uni-ulm.de)

**Supplemental Table 1: Functionality and pain scores during the follow-ups (mean, standard error)**

|  | | **Male** | | **Female** | |
| --- | --- | --- | --- | --- | --- |
| **Variables** | **Total** | **hip** | **knee** | **hip** | **knee** |
| FFbH at baseline (N=686) | 67.26 (0.6) | 68.7 (1.22) | 75.63 (1.47) | 61.25 (1.01) | 67.55 (1.01) |
| FFbH after 6 months (N=532) | 79.90 (0.66) | 83.23 (1.39) | 82.89 (1.61) | 78.54 (1.23) | 77.19 (1.11) |
| FFbH after 12 months (N=534) | 80.53 (0.71) | 84.50 (1.39) | 82.45 (1.95) | 80.29 (1.23) | 76.64 (1.26) |
| FFbH after 60 months (N=590) | 78.90 (0.79) | 85.60 (1.44) | 83.15 (1.99) | 78.09 (1.34) | 72.79 (1.45) |
| VAS-Pain at baseline (N=799) | 28.49 (0.61) | 33.58 (1.36) | 29.98 (1.41) | 27.18 (1.11) | 25.34 (0.99) |
| VAS-Pain after 6 months (N=478) | 82.89 (1.01) | 87.16 (1.80) | 75.52 (3.05) | 88.17 (1.71) | 78.47 (1.80) |
| VAS-Pain after 12 months (N=408) | 85.88 (1.00) | 87.63 (1.76) | 80.09 (3.47) | 88.91 (1.53) | 83.56 (2.01) |
| VAS-Pain after 60 months (N=603) | 65.66 (1.86) | 72.22 (3.25) | 57.60 (5.47) | 63.70 (4.37) | 65.63 (2.78) |
| WOMAC Score at Baseline (N=650) | 54.93 (0.59) | 52.64 (1.31) | 48.46 (1.65) | 58.72 (1.02) | 56.03 (0.91) |
| WOMAC Score after 6 months (N=468) | 26.41 (0.85) | 21.90 (1.69) | 31.51 (2.18) | 22.43 (1.57) | 30.67 (1.42) |
| WOMAC Score after 12 months (N=459) | 23.3 (0.92) | 19.77 (1.61) | 27.26 (2.5) | 19.14 (1.53) | 28.9 (1.81) |
| WOMAC Score after 60 months (N=504) | 22.83 (0.92) | 18.17 (1.64) | 25.71 (2.93) | 20.67 (1.56) | 28.17 (1.73) |
| WOMAC Pain Score at Baseline (N=648) | 11.78 (0.14) | 10.68 (0.31) | 10.64 (0.34) | 12.27 (0.23) | 12.61 (0.21) |
| WOMAC Pain Score after 6 months (N=564) | 4.89 (0.17) | 3.76 (0.33) | 5.86 (0.46) | 3.77 (0.28) | 6.28 (0.31) |
| WOMAC Pain Score after 12 months (N=562) | 4.56 (0.18) | 3.84 (0.33) | 5.38 (0.50) | 3.64 (0.31) | 5.65 (0.34) |
| WOMAC Pain Score after 60 months (N=582) | 4.23 (0.19) | 3.51 (0.43) | 5.26 (0.54) | 3.29 (0.3) | 5.21 (0.34) |
| WOMAC Functionality Score at Baseline (N=628) | 38.59 (0.45) | 37.30 (0.99) | 33.34 (1.32) | 41.75 (0.77) | 38.95 (0.71) |
| WOMAC Functionality Score after 6 months (N=484) | 18.76 (0.61) | 16.09 (1.25) | 22.22 (1.59) | 16.23 (1.16) | 21.26 (1.01) |
| WOMAC Functionality Score after 12 months (N=482) | 16.61 (0.65) | 14.58 (1.22) | 19.02 (1.80) | 13.60 (1.07) | 20.24 (1.24) |
| WOMAC Functionality Score after 60 months (N=548) | 16.87 (0.66) | 13.09 (1.13) | 20.15 (2.24) | 15.86 (1.18) | 19.82 (1.17) |

WOMAC=Western Ontario and McMaster University Osteoarthritis Index; VAS=visual analogue scale; FFbH =Hannover Functionality Status Questionnaire; N= number of observations (may not add up to total because of missing items for some variables)

**Supplemental Table 2: Correlation between function and pain within the two sets of instruments (FFbH and VAS-pain, and WOMAC functionality score and WOMAC pain score) at baseline, the change between baseline and month 6, change between month 6 and month 12, and change between month 12 and month 60**

|  | **Between  FFbH and VAS-pain** | **Between WOMAC functionality score and pain score** |
| --- | --- | --- |
| **Time points** | **Correlation coefficients (95% confidence intervals)** | |
| Baseline | 0.201 (0.128, 0.272) | 0.693 (0.650, 0.732) |
| Change between baseline and month 6 | 0.149 (0.047, 0.248) | 0.726 (0.674, 0.77) |
| Change between month 6 and month 12 | 0.065 (-0.061, 0.189) | 0.606 (0.530, 0.671) |
| Change between month 12 and month 60 | 0.060 (-0.056, 0.174) | 0.678 (0.616, 0.731) |
