# Supplementary material for: Five years’ trajectories of functionality and pain in patients after hip or knee replacement and association with long-term patient survival
Source: Sci Rep. 2020 Sep 1;10:14388. doi: 10.1038/s41598-020-71277-3 (PMC7463234; doi:10.1038/s41598-020-71277-3)
Supplement: Supplementary file 2 — Supplementary file2 [file 41598_2020_71277_MOESM2_ESM.docx]

**Appendix Methods**

Observations with the missing baseline measurements of functionality and pain were excluded. Therefore, for VAS and FFbH N=675 subjects remained in analyses and for WOMAC sub-scales N=625. Additionally, to account for missing values (assuming missing at random) over the course of time and to adjust for general measurement errors, a two-dimensional mixed-effects model under the assumption of a piecewise linear trajectory was conducted for pain and functionality in landmark datasets, only including patients alive at the landmark time of interest. The two-dimensional approach resulted from the simultaneous estimation of our variables of interest. Our variables were estimated by time only and did not include any other covariates. Breakpoints of the partwise linear approach were chosen due to the data structure. Since the data only provided information on pain and function during the follow-up times we chose those times as Breakpoints. The exact times were therefore determined as 6 months and 12 months after follow up. We were unable to model a 60-month breakpoint since there are no observations later in time. All the above led to model (1), where the β_j_ represent fixed effects and the b_ji_ represent random effects of patient i. We did not assume any explicit dependency structure in the random effects.

$$(\begin{aligned} Pain \\ Function \end{aligned})=\begin{aligned} (\beta_{0}+b_{0i})+(\beta_{1}+b_{1i})t+(\beta_{2}+b_{2i})(t-6)1_{t>6}+(\beta_{3}+b_{3i})(t-12)1_{t>12}+\epsilon_{1i} \\ (\beta_{4}+b_{4i})+(\beta_{5}+b_{5i})t+(\beta_{6}+b_{6i})(t-6)1_{t>6}+(\beta_{7}+b_{7i})(t-12)1_{t>12}+\epsilon_{2i} \end{aligned}(1)$$

The landmark approach following these estimations, calculates a single Cox-model for every landmark time, including only those patients still alive at the time. The estimated current slopes from model (1) were then included in the corresponding landmark Cox model with the landmark times 6, 12, and 60 chosen according to the data structure and the prior linear model. [11,12] In more detail the slopes of each patient were calculated by adding up all prior slopes. An Example may be the slope of Pain at time 12, which was estimated by (β_1_+b_1i_+ β_2_+b_2i_ ). The Cox-models for each landmark time in this analysis included the baseline values of functionality and pain, and the following baseline covariates were considered: age, gender, diabetes, cholesterol, uric acid, heart insufficiency, hypertension, overweight, smoking status, localization of OA, and secondary OA as covariates for overall survival, and the slopes during the previous time-interval for pain and functionality, separately estimated for the two approaches. The adjustment for covariates followed the former analyses [9] in which a backward selection approach was applied, starting with a large number of covariates and keeping variables with p-values less than 0.10. Variable selection was therefore not part of the presented analysis. Our exact Cox Model for each landmark point is presented in model (2), where the baseline values of pain and functionality are represented by B_i1_ and B_i2_. S_i1_ and S_i2_ are the slopes just prior to the landmark time and Z_i_  are all other previously mentioned covariates.

$$\alpha(t\mid B_{i1},B_{i2},S_{i1},S_{i2},Z_{i})=\alpha_{0}(t)\exp(\beta_{0}B_{i1}+\beta_{1}B_{i2}+\beta_{2}S_{i1}+\beta_{3}S_{i2}+\beta^{t}Z_{i})(2)$$

Hazard ratios (HR) for the improvement in functionality or pain were estimated from the Cox models. The HRs with 95% confidence intervals (CI) and additional p-values are shown for all 6 resulting sub models (3 time-points for 2 approaches). No adjustment for multiple testing was performed. All analyses were performed using SAS 9.4 (SAS Institute Inc., Cary, NC, USA).
